# Impact of periodontal conditions on the quality of life of pregnant women: a cross-sectional study

**DOI:** 10.1186/s12955-015-0267-8

**Published:** 2015-05-28

**Authors:** Hai-Xia Lu, Wei Xu, May Chun Mei Wong, Tian-You Wei, Xi-Ping Feng

**Affiliations:** Department of Preventive Dentistry, Ninth People’s Hospital, Shanghai Jiao Tong University, School of Medicine, Shanghai Key Laboratory of Stomatology, Shanghai, China; Department of Preventive Dentistry, Shanghai Municipal Hospital for Oral Health, Shanghai, China; Dental Public Health, Faculty of Dentistry, University of Hong Kong, Hong Kong, China

**Keywords:** Pregnant women, Oral health-related quality of life, Oral health impact profile, Periodontal disease, Tooth loss

## Abstract

**Background:**

Studies have been rarely conducted to provide a comprehensive perspective of pregnant women with the intention to investigate the relationships between periodontal conditions and oral health-related quality of life (OHRQoL). As such, this study aimed to describe the OHRQoL of pregnant women in Shanghai, China and to investigate the relationships between periodontal conditions and OHRQoL of pregnant women.

**Methods:**

A cross-sectional study was conducted amongst pregnant women in all stages of pregnancy in Shanghai, China. Clinical examinations were performed to assess periodontal conditions, including tooth loss, visible plaque index, bleeding on probing, probing pocket depth and clinical attachment level. The OHRQoL of pregnant women was determined using the Oral Health Impact Profile (OHIP-14, Chinese version). Information regarding maternal characteristics, socio-demographic background and health-related behaviours was also obtained from the participants through the structured questionnaires.

**Results:**

A total of 512 pregnant women (mean age = 27.3 ± 4.0 years)participated in the survey,giving a response rate of 91.4 %. The mean gestational age was 19 weeks (SD = 8.2). The mean and the median OHIP-14 scores were 7.92 (SD = 6.84) and 6, respectively. The mean number of negative impact items (extent) was 0.20 (SD = 0.82). Approximately 10 % of pregnant women reported at least one item with ‘fairly often’ or ‘very often’ (prevalence). Results of multivariable analyses showed that periodontal conditions was not significantly associated with three scoring formats of OHRQoL (severity, extent and prevalence of impact) after adjustment for pregnancy-related variables and possible confounders (all p > 0.05). However, frequency of nausea-vomiting was found to be significantly associated with severity of impacts (*p* = 0.012). Utilization of dental services, age and tooth loss were the significant variables to the extent of negative impacts (all p < 0.05). While no significant variable was related with prevalence of negative impacts (p > 0.05).

**Conclusion:**

Pregnant women with different trimesters showed similar impact of oral disease on their OHRQoL in Shanghai, China. Periodontal health status have no impact on their OHRQoL in the fully adjusted models. Their OHRQoL was associated with early pregnancy reaction, utilisation of dental services, age and tooth loss.

## Background

During pregnancy, immunoresponsiveness and inflammatory response mediators have been altered because of increased progesterone and oestrogen levels; changes in these hormone levels, as well as in oral hygiene habit and lifestyle, may result in increased susceptibility of pregnant women to periodontal disease [1]. The relationships between pregnancy and periodontal health status have been well documented [1–10]. For instance, epidemiological studies have shown that the prevalence of gingivitis during pregnancy, termed as pregnancy gingivitis, varies from 35 to 100 %; pregnancy gingivitis is characterised by gingival erythema, hyperplasia and bleeding [2–4, 11]. Periodontal disease is usually asymptomatic from gingivitis to periodontitis, but this disease is also characterised by clinical signs and symptoms, including bleeding, tooth shift or loss, periodontal abscesses or halitosis [12]. Periodontal disease during pregnancy affects not only maternal oral health but also foetal growth with increased risk of subsequent preterm birth or low birth weight [13, 14]. Moreover, treatment (scaling and root planning) for maternal periodontal disease may not effectively reducec adverse pregnancy outcomes [15].

In addition to assessment of health status through clinical measures, patient-based assessment of health status is essential to evaluate health. The impact of diseases on the function or psychosocial well-being of a person has been commonly evaluated and defined as health-related quality of life. Indeed, the assessment of health-related quality of life has been considered as an indispensable part of evaluation programs in research, public health and clinical purposes [16] in response to Locker’s proposition that a disease-based biomedical approach should be changed to a patient-based biopsychosocial approach in health care [17]. A complementary perspective on functional, social and psychological consequences of oral diseases (such as periodontal disease) during pregnancy is necessary to plan and assess the dental care provided for pregnant women; this perspective is also important to address pregnant women’s needs and concerns [18].

Thus far, very few studies on the oral health-related quality of life (OHRQoL) of pregnant women have been conducted [18–21]. In one study, the oral pain of low-income pregnant Brazilian women negatively affects their OHRQoL [19]. In another study conducted in rural Indian pregnant women, the impact of dental caries and periodontal health status on OHRQoL were investigated. Periodontal disease and previous pregnancies were associated with poorer OHRQoL [20, 21]. However, contradictory findings were reported in Uganda and Argentine pregnant women, which found that periodontal status had no impact on their OHRQoL while tooth loss was strongly associated with poorer OHRQoL [18, 22]. However, the studies conducted in Indian and Uganda adopted community probe index (CPI) with partial mouth examination to assess periodontal health status [18, 20, 21], which may not provide accurate information as there is a consensus that the full mouth assessment rather than partial mouth examination would optimally examine periodontal conditions [23]. Although the study in Argentine adopted periodontal full mouth assessment, the findings were based on small samle size [22]. Hence, periodontal full-mouth assessment with large scale study should be applied to investigate the relationships between periodontal conditions and OHRQoL of pregnant women.

Over the past decades, different indicators and measures have been developed to assess the OHRQoL of adults. Oral health impact profile (OHIP) is amongst the commonly used OHRQoL measures. OHIP is based on Locker’s oral health-related model [24]. The Chinese version of OHIP was translated and validated in 2002 [25]. Therefore, Chinese researchers utilised this validated measure to evaluate the impact of oral diseases or disorders on the OHRQoL of adults in China. However, no study has been performed to assess the OHRQoL of pregnant women. This study aimed to describe the OHRQoL of pregnant women in Shanghai, China and to investigate the relationships between periodontal conditions and OHRQoL of pregnant women after adjustment for pregnancy-related variables and socio-demographic background.

## Materials and methods

### Study population

A cross-sectional study was conducted of pregnant women in all stages of pregnancy in Shanghai, China. In Shanghai, 17 maternal and child care service centres at a county level have been established; amongst these centres, 11 are located in urban districts and 6 are found in rural counties. With limited resources, three centres (two urban and one rural maternal and child care service centres) were randomly selected. In each selected care service centre, pregnant women who attended antenatal checkup were consecutively recruited from October 2012 to March 2013.

Ethical approval was obtained from the Institutional Review Board of the Ninth People’s Hospital, Shanghai Jiao Tong University School of Medicine prior to the implementation of the study. Written informed consent was obtained from the participants, as appropriate.

Sample size was calculated. With reference to a previous study involving pregnant women in India [21], the standard deviation of OHIP-14 scores was approximately 7.4. The 95 % confidence interval was set to be 0.7 on both sides of the mean OHIP-14 score. The computed minimum sample size was 429. With a response rate of 85 %, at least 505 pregnant women were needed. When the sample size was determined based on the analytical aims of the present study, totally 17 independent variables were considered in the multivariable analyses. As a rule of thumb, a number of 10–20 observations per variable are necessary to avoid computational difficulties for multivariable analysis. The observations for this study were 170–340, which was considerably less than 505 which we established for descriptive purposes of the OHIP measures. Therefore, a sample size of 505 pregnant women has covered the descriptive and analytical purposes.

### Data collection

Data were obtained by facilitating a clinical examination and conducting a questionnaire survey. The participants were clinically examined in terms of tooth loss and periodontal health status. Accumulated tooth loss was obtained by counting the number of tooth loss due to any reason except third molars. All teeth present in the mouth (including third molars) were subjected to periodontal examination. Visible plaque index (VPI) was respectively scored as 0 or 1 corresponding to the absence or the presence of bacterial plaque on two surfaces per tooth; the percentage of the surfaces with plaque was also calculated [26]. Bleeding on probing (BOP) was evaluated using a dichotomous index respectively scored as 0 or 1 corresponding to the absence or the presence of bleeding on two surfaces per tooth; the percentage of the surfaces with bleeding was also determined [27]. Probing pocket depth (PPD; measured from the gingival margin to the total probing depth) was assessed at six sites per tooth (mesiobuccal, buccal, distobuccal, distolingual, lingual and mesiolingual) by using the following scores: 0 = no probing pocket; 1 = 4 mm to 5 mm depth of probing pocket; and 2 = higher than 6 mm depth of probing pocket [27]. Clinical attachment level (CAL; measured from the cemento-enamel junction to the total probing depth) was scored using four measures: 0 = 0 mm to 3 mm of CAL; 1 = 4 mm to 5 mm, 2 = 6 mm to 8 mm; 3 = 9 mm to 11 mm; and 4 = higher than 11 mm. Examinations were conducted in each maternal and child care service centre. The participants were examined by two trained and calibrated examiners who used a disposable mouth mirror attached to an intraoral LED light and lightweight CPI probes with a ball-tip end diameter of 0.5 mm. Approximately 10 % of these participants were then re-examined to monitor inter-examiner reproducibility As measured using Kappa statistics, the inter-examiner reliabilities of VPI, BOP, PPD and CAL were 0.86 (95 % CI: 0.81-0.91), 0.72 (95 % CI: 0.66-0.78), 0.75 (95 % CI: 0.71-0.79) and 0.70 (95 % CI: 0.64-0.76), respectively.

After clinical examination was conducted, the participants were instructed to self-complete a structured questionnaire to obtain information pertaining to maternal characteristics (trimester, previous pregnancy history and self-reported systemic disease) and socio-demographic background (age, marriage status, location, birthplace, educational attainment, monthly household income level, dental insurance coverage). Health-related behaviors (such as utilisation of dental services and the frequency of nausea-vomiting) were also collected. The utilisation of dental services was determined by asking participants whether or not they underwent a dental visit during pregnancy. If so, the reason for dental visit was a regular dental checkup or only due to problem arose. Self-perceived oral health measures during pregnancy (perceived oral health status, perceived oral health impact on daily life and perceived dental treatment need) was also determined.

The OHRQoL of the participants was assessed using the Chinese version of the short form of OHIP-14 [25]. These participants were asked how often they experienced the impact because of problems with teeth, mouth, or dentures (related to 14 items) since these women have become pregnant. Responses to each item were rated on a five-point Likert scale and coded as follows: 4 = “very often”; 3 = “fairly often”; 2 = “occasionally”; 1 = “hardly ever”; and 0 = “never” [28]. Three scoring formats were adopted to assess the level of impact on quality of life [29]. The unweighted OHIP-14 score was calculated as the sum of the response codes of the 14 items. The total OHIP-14 score was designated as the “severity” of impacts. The extent of negative impacts indicating the number of items reported ‘fairly often’ or ‘very often’ was generated. The prevalence (percentage) of the participants with negative impacts (reporting at least one item with ‘fairly often’ or ‘very often’) was also determined.

### Data analysis

The internal consistency of OHIP-14 in this population was assessed using Cronbach’s α. Test-retest correlation coefficients were obtained to assess the reliability of OHIP-14 by using a time interval of 2 weeks between the administration of the two questionnaires amongst 51 participants. Construct validity was investigated by determining whether or not the OHIP-14 score was associated with perceived oral health status, perceived oral health impact on daily life and perceived dental treatment need.

The descriptive statistics (mean, standard deviation and percentage) of the severity, extent and prevalence of the impacts of oral disorders was presented. Independent variables included the clinical periodontal conditions, socio-demographic background, pregnancy-related variables of pregnant women. Bivariate relationships between each independent variable and the prevalence of negative impacts reported as “fairly often” or “very often” were assessed using Chi-square tests. Mann–Whitney U tests or Kruskal-Wallis one-way ANOVA were performed to compare the distribution of the severity and the extent of impacts amongst different groups of each independent variable because data were not normally distributed.

**Fig. 1:**
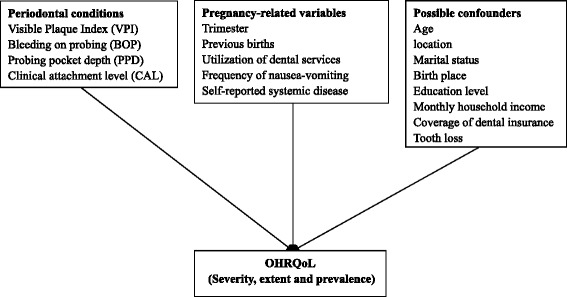
Theoretical framework of the relationships between independent variables and OHRQoL of pregnant women

Multivariable analyses were conducted to evaluate the factors associated with the three scoring formats of the OHRQoL measures of the participants. Multiple negative binomial or Poisson regressions, whichever was appropriate (deviance ratio close to 1), were conducted to investigate the factors correlated with the severity and the extent of impacts; The prevalence of negative impacts (‘fairly often’ or ‘very often’) was modelled using multiple logistic regression. The theoretical framework for the present study was constructed (Fig. 1). The independent variables in the model were selected from those reported to have a direct relationship with OHRQoL described in previous studies [16, 18, 20–22]; these independent variables were then grouped into clinical periodontal conditions, pregnancy-related variables and non-pregnancy-related variables. As shown in Fig. 1, pregnancy-related variables included trimesters, previous births, frequency of nausea-vomiting, utilization of dental services during pregnancy and self-reported systematic disease. Non-pregnancy-related variables including tooth loss and socio-demographic factors were considered as possible confounders. In Model 1, only clinical periodontal conditions were entered in the multivariable analyses. In Model 2, multivariable analyses were performed for periodontal conditions after adjustment for pregnancy-related variables. In Model 3, multivariable analyses were performed after adjustment for pregnancy-related variables and possible confounders. Data were analysed using SPSS 22.0 (IBM Corp., Armonk, NY, USA). In all of the statistical tests, significance level was set at 0.05.

## Results

### Description of the study population

A total of 560 pregnant women from three maternal and child care service centres were invited to participate in this study. A total of 48 pregnant women refused to participate in the survey or could not tolerate clinical oral examinations. Finally, only 512 pregnant women (mean age = 27.3 ± 4.0, median = 27, range = 18 years to 42 years) participated in the survey; thus, a response rate of 91.4 % was obtained. The mean gestational age was 19 weeks (SD = 8.2, range = 5 weeks to 40 weeks) with 38.9 %, 35.7 % and 25.4 % in the first trimester (1 week to 14 weeks), second trimester (15 weeks to 25 weeks) and third trimester (26 weeks to 40 weeks), respectively. The majority of the participants (95.1 %) was married and and 79.6 % were in their first pregnancy. Approximately two-thirds (60.5 %) of these women were urban residents, and approximately half (44.5 %) of these women were born in Shanghai. More than half of the participants presented educational attainment of matriculation or bachelor degree and received a monthly household income of RMB 6000 and higher (55.1 and 52.3 %, respectively). Approximately 60 % of the participants were covered by dental insurance. Almost 50 % of the participants suffered from nausea-vomiting twice or more during pregnancy. Approximately 4 % of the pregnant women utilised dental services during pregnancy with any reason (1.2 % for regular dental checkup and 2.9 % for oral problems). 16.8 % of them self-reported they suffered from systematic disease.

Around 20 % of the participants lost their teeth because of any reason.

### Periodontal conditions

Periodontal conditions of the studied pregnant women according to their trimester are presented in Table 1. Less than half (44.7 %) of the participants showed >50 % of the tooth sites with visible plaque. More than 50 % of the participants showed >25 % of tooth sites with bleeding and PPD ≥ 4 mm. Less than one-third (27.1 %) of the participants exhibited tooth sites with CAL ≥ 4 mm. The participants with a higher trimester yielded significantly more tooth sites with bleeding and PPD ≥ 4 mm than those in other trimester (all p < 0.05).

**Table 1:** Periodontal conditions of the studied pregnant women according to their trimesters

	First trimester	Second trimester	Third trimester	p value	Total
Variables	n (%)	n (%)	n (%)		n (%)
VPI				0.324	
<50 % site with plaque	118 (59.3)	98 (53.6)	67 (51.5)		283 (55.3)
50 % + site with plaque	81 (40.7)	85 (46.4)	63 (48.5)		229 (44.7)
GBI				0.001	
<25 % site with bleeding	114 (57.3)	71 (38.8)	55 (42.3)		240 (46.9)
25 % + site with bleeding	85 (42.7)	112 (61.2)	75 (57.7)		272 (53.1)
PPD				<0.001	
<25 % site with PPD > =4	128 (64.3)	71 (38.8)	52 (40.0)		251 (49.0)
25 % + site with PPD > =4	71 (35.7)	112 (61.2)	78 (60.0)		261 (51.0)
CAL				0.218	
0 % site with CAL > =4	146 (73.4)	126 (68.9)	101 (77.7)		373 (72.9)
0 % + site with CAL > =4	53 (26.6)	57 (31.1)	29 (22.3)		139 (27.1)

### OHRQoL

The severity, extent and prevalence of impacts accordin to the individual OHIP-14 items are summaried in Table 2. The mean and the median OHIP-14 scores were 7.92 (SD = 6.84, range = 0 to 38) and 6 (interquartile range = 11), respectively. The mean number of the items with negative impact (extent) was 0.20 (SD = 0.82, range = 0 to 10). About 10 % of the pregnant women (10.2 %) reported at least one item with ‘fairly often’ or ‘very often’ (prevalence). The most commonly reported impacts with ‘fairly often’ or ‘very often’ were within the domains of “functional limitation”, “physical pain” and “psychological discomfort”. The item “taste worse” (3.5 %) was the item with the highest impacts with ‘fairly often’ or ‘very often’, followed by “painful aching” (2.9 %), “uncomfortable to eat” (2.7 %) and “self-conscious” (2.7 %), with the highest mean scores for those items (range = 0.75 to 0.95). The proportion of the participants who reported impacts with ‘fairly often’ or ‘very often’ of the items in the domains of “social disability” and “handicap” was the lowest (0 to 0.4 %).

**Table 2:** The severity, extent and prevalence of impacts according to the individual OHIP-14 items

	Severity^a^	Extent^b^	Prevalence^c^
	Mean (SD)	Mean (SD)	n (%)
Function limitation			
Q1 Trouble pronouncing	0.43 (0.71)	0.01 (0.11)	6 (1.2)
Q2 Taste worse	0.82 (0.88)	0.04 (0.18)	18 (3.5)
Physical pain			
Q3 Painful aching	0.95 (0.84)	0.03 (0.17)	15 (2.9)
Q4 Uncomfortable to eat	0.92 (0.82)	0.03 (0.16)	14 (2.7)
Psychological discomfort			
Q5 Self-conscious	0.75 (0.84)	0.03 (0.16)	14 (2.7)
Q6 Being tense	0.61 (0.78)	0.02 (0.13)	9 (1.8)
Physical disability			
Q7 Diet unsatisfactory	0.63 (0.78)	0.01 (0.12)	7 (1.4)
Q8 Interrupt meals	0.54 (0.71)	0.01 (0.12)	7 (1.4)
Psychological disability			
Q9 Difficult relax	0.51 (0.65)	<0.01 (0.08)	3 (0.6)
Q10 Been embarrassed	0.57 (0.72)	<0.01 (0.10)	5 (1.0)
Social disability			
Q11 Irritable with others	0.35 (0.59)	<0.01 (0.04)	1 (0.2)
Q12 Difficulty doing jobs	0.28 (0.50)	<0.01 (0.00)	0 (0.0)
Handicap			
Q13 Life unsatisfying	0.35 (0.59)	<0.01 (0.06)	2 (0.4)
Q14 Unable to function	0.24 (0.49)	<0.01 (0.04)	1 (0.2)
Total	7.92 (6.84)	0.20 (0.82)	52 (10.2)

^a^OHIP-14 score

^b^The number of items reported ‘fairly often’ or ‘very often’

^c^The percentage of the participants report at least one item with ‘fairly often’ or ‘very often’

The reliability of the OHIP-14 score was assessed, and Cronbach’s α coefficient (α = 0.91) indicating the OHIP-14 score showed good internal consistency. The test-retest correlation of OHIP-14, as measured using Pearson’s correlation coefficient, was 0.78. Furthermore, the good construct validity of the OHIP-14 score was supported by the significant association of mean scores with perceived oral health status, impact of oral health on daily life and dental treatment need (p < 0.05).

### Bivariate associations between periodontal conditions, pregnancy-related variables, socio-demographic background, and OHRQoL

The bivariate association between the severity, extent and prevalence of impacts and periodontal conditions is shown in Table 3. CAL was significantly associated with severity scores (*p* = 0.025). The pregnant women who suffered from >25 % of sites with bleeding exhibited a significantly higher extent and prevalence of negative impacts (*p* = 0.032 and *p* = 0.031, respectively).

**Table 3:** The severity, extent and prevalence of impacts on the studied pregnant women according to their periodontal conditions

	Severity		p value*	Extent		p value*	Prevalence		p value
Variables	Mean	SD		Mean	SD		n	%	
VPI			0.588			0.691			0.711
<50 % site with plaque	8.08	6.89		0.22	0.92		30	10.6	
50 % + site with plaque	7.73	6.78		0.17	0.68		22	9.6	
BOP			0.080			0.032			0.031
<25 % site with bleeding	7.49	6.92		0.16	0.83		17	7.1	
25 % + site with bleeding	8.31	6.75		0.24	0.81		35	12.9	
PD			0.595			0.431			0.466
<25 % site with PD > =4	7.7	6.69		0.17	0.81		23	9.2	
25 % + site with PD > =4	8.14	6.98		0.23	0.83		29	11.1	
CAL			0.025			0.057			0.053
0 % site with CAL > =4	7.51	6.69		0.18	0.86		32	8.6	
0 % + site with CAL > =4	9.02	7.13		0.24	0.71		20	14.4	

*p value was computed from Mann–Whitney *U* test or Kruskal Wallis one-way ANOVA test

Table 4 presents the relationships between the severity, extent and prevalence of impacts and pregnancy-related variables amongst pregnant women. The frequency of nausea-vomiting during pregnancy was significantly related to the severity of impacts (p < 0.001). However, in the bivariate analyses, no significant association was observed between trimesters, previous births, utilization of dental services, self-reported systemic disease, and three scoring formats of the OHRQoL measures (all p > 0.05).

**Table 4:** The severity, extent and prevalence of impacts on the studied pregnant women according to their pregnancy-related variables

		Severity		p value*	Extent		p value*	Prevalence		p value
Variables	n (%)	Mean	SD		Mean	SD		n	%	
Trimester				0.082			0.897			0.903
First	199 (38.9)	8.29	6.30		0.18	0.72		19	9.5	
Second	183 (35.7)	8.20	7.46		0.22	0.82		20	10.9	
Third	130 (25.4)	6.98	6.67		0.20	0.97		13	10	
Previous births^a^				0.628			0.360			0.341
No	406 (79.6)	7.88	6.92		0.19	0.84		38	9.4	
One or more	104 (20.4)	8.00	6.46		0.20	0.72		13	12.5	
Frequency of nausea-vomiting				0.001			0.157			0.172
Once or less	259 (50.6)	6.93	6.69		0.21	0.99		22	8.5	
Twice	154 (30.1)	8.61	6.77		0.13	0.45		15	9.7	
Three times or more	99 (19.3)	9.44	6.99		0.28	0.77		15	15.2	
Utilization of dental services				0.750			0.380			0.377
No	491 (95.9)	7.87	6.80		0.17	0.41		1	16.7	
Yes	21 (4.1)	9.19	7.79		0.60	1.81		3	20.0	
Self-reported systematic disease				0.256			0.450			0.375
No	86 (16.8)	8.08	6.89		0.21	0.89		41	9.6	
Yes	426 (83.2)	7.15	6.54		0.14	0.38		11	12.8	

^a^Variables with some missing data

*p value was computed from Mann–Whitney *U* test or Kruskal Wallis one-way ANOVA test

Table 5 presents the relationships between the severity, extent and prevalence of impacts and possible confounders amongst pregnant women. The participants who were rural residents and born outside Shanghai exhibited significantly higher severity, extent and prevalence of impacts than those who reside in urban areas and born in Shanghai (all p < 0.05). The monthly household income of the participants was significantly associated with severity scores (*p* = 0.023); educational attainment was also related to the extent of negative impacts (*p* = 0.043).

**Table 5:** The severity, extent and prevalence of impacts on the studied pregnant women according to possible confounders

		Severity		p value*	Extent		p value*	Prevalence		p value
Variables	n (%)	Mean	SD		Mean	SD		n	%	
Age (Years)				0.074			0.552			0.545
25 or below	168 (32.8)	8.00	6.71		0.20	0.61		21	12.5	
26-30	246 (48.0)	8.02	6.95		0.20	0.89		23	9.3	
31-35	83 (16.2)	6.73	6.34		0.16	0.82		6	7.2	
36 or above	15 (2.9)	12.00	7.96		0.47	1.55		2	13.3	
Marital status				0.639			0.096			0.095
Married	487 (95.1)	7.91	6.87		0.19	0.83		47	9.7	
Single	25 (4.9)	8.16	6.28		0.32	0.75		5	20	
Location				0.039			0.011			0.011
Urban	310 (60.5)	7.54	6.93		0.15	0.81		23	7.4	
Rural	202 (39.5)	8.52	6.67		0.27	0.84		29	14.4	
Birth place				0.022			0.007			0.007
Shanghai	228 (44.5)	7.22	6.75		0.13	0.76		14	6.1	
Other places	284 (55.5)	8.49	6.76		0.26	0.87		38	13.4	
Education level				0.937			0.043			0.051
Higher secondary school or below	230 (44.9)	7.94	6.80		0.25	0.80		30	13	
Matriculation or bachelor degree or above	282 (55.1)	7.91	6.88		0.16	0.84		22	7.8	
Monthly household income^a^				0.023			0.070			0.070
Less than RMB6000	243 (47.7)	8.65	7.01		0.24	0.85		31	12.8	
RMB6000 and more	266 (52.3)	7.30	6.65		0.16	0.81		21	7.9	
Coverage of dental insurance^a^				0.589			0.114			0.220
No	206 (40.2)	7.98	6.56		0.23	0.81		26	12.6	
Yes	301 (58.8)	7.89	7.07		0.18	0.84		25	8.3	
Tooth loss				0.109			0.387			0.422
0 teeth missing	415 (81.1)	7.66	6.66		0.18	0.77		40	9.6	
1+ teeth missing	97 (18.9)	9.07	7.48		0.29	1.01		12	12.4	

^a^Variables with some missing data

*p value was computed from Mann–Whitney *U* test or Kruskal Wallis one-way ANOVA test

### Multivariable analyses

Negative binomial regression models (Models 1–3) of the relationships between independent variables and severity of impacts are shown in Table 6. In Model 1, no variable was significantly associated with severity of impacts. In model 2, CAL and Frequency of nausea-vomiting were found to be significant after adjustment for pregnancy-related variables. In model 3, only frequency of nausea-vomiting still showed the sifnificance after adjustment for possible confounders while significant association between CAL and severity of impacts was no longer observed. The result of model 3 showed that pregnant women experiencing more frequent nausea-vomiting exhibited significantly higher severity scores (twice: IRR = 1.24; three times or more: IRR = 1.42; *p* = 0.012) than those who vomited once or less, indicating poorer OHRQoL.

**Table 6:** Results of negative binomial regressions for the severity of impacts on the studied pregnant women

Variables	Model 1^a^			Model 2^b^			Model 3^c^		
	IRR	95 % CI	P value	IRR	95 % CI	P value	IRR	95 % CI	P value
Model 1									
VPI			0.149			0.154			0.302
<50 % site with plaque vs. 50 % + site with plaque	/	/		/	/		/	/	
BOP			0.252			0.290			0.406
<25 % site with bleeding vs. 25 % + site with bleeding	/	/		/	/		/	/	
PPD			0.924			0.755			0.864
<25 % site with PD > =4 vs. 25 % + site with PD > =4	/	/		/	/		/	/	
CAL			0.062			0.025			0.138
0 % site with CAL > =4 vs. 0 % + site with CAL > =4	/	/		1.29	1.03-1.61		/	/	
Model 2									
Trimesters						0.333			0.473
First vs. Second				/	/		/	/	
vs. Third				/	/		/	/	
Previous births						0.917			0.977
None vs. One or more				/	/		/	/	
Utilization of dental services						0.366			0.605
No vs. Yes				/	/		/	/	
Frequency of nausea-vomiting						0.011			0.012
Once or less vs. Twice				1.28	1.03-1.58		1.24	1.00-1.55	
vs. Three times or more				1.4	1.09-1.79		1.42	1.10-1.82	
Self-repored systematic disease						0.186			0.250
No vs. Yes				/	/		/	/	
Model 3									
Age (Years)									0.431
25 or below vs. 26-30							/	/	
vs. 31-35							/	/	
vs. 36 or above							/	/	
Location									0.861
Urban vs. Rural							/	/	
Marital status									0.616
Married vs. Single							/	/	
Birth place									0.199
Shanghai vs. Other places							/	/	
Education level									0.186
Higher secondary school or below vs. Matriculation or bachelor degree or above							/	/	
Monthly household income									0.872
Less than RMB 6000 vs. RMB6000 or more							/	/	
Coverage of dental insurance									0.058
No vs. Yes							/	/	
Tooth loss									0.119
0 teeth missing vs. 1+ teeth missing							/	/	

^a^Likelihood Ratio *χ*2 = 5.8, df = 4, *p* =0.211; Deviance ratios = 1.07

^b^Likelihood Ratio *χ*2 = 19.1, df = 11, *p* =0.059; Deviance ratios = 1.06

^c^Likelihood Ratio *χ*2 = 30.8, df = 22, *p* =0.100; Deviance ratios = 1.06

Model 1: Negative binomial regression for periodontal condition variables

Model 2: Negative binomial regression for periodontal condition variables after adjustment for pregnancy-related variables

Model 3: Negative binomial regression for periodontal condition variables after adjustment for pregnancy-related variables and possible confounders

Regarding the extent of negative impacts, Poisson regression models (Models 1–3) are shown in Table 7. In the final model (Model 3), three variables (utilization of dental services, age and tooth loss) showed the sifnificance after adjustment for possible confounders (p < 0.05) while none of periodontal conditions variables was significantly associated with the extent of negative impacts. Pregnant women who utilised dental services during pregnancy (IRR = 2.07, *p* = 0.044), who aged 36 years or above (IRR = 5.06, *p* = 0.009) and who had one or more missing teeth (IRR = 1.85, *p* = 0.011) showed a significantly higher mean number of the items reporting ‘fairly often’ or ‘very often’ (extent of negative impacts), indicating poorer OHRQoL.

**Table 7:** Results of Poisson regression for the extent of negative impacts (‘fairly often’ or ‘very often’) on the studied pregnant women

Variables	Model 1^a^			Model 2^b^			Model 3^c^		
	IRR	95 % CI	P value	IRR	95 % CI	P value	IRR	95 % CI	P value
Model 1									
VPI			0.011			0.057			0.056
<50 % site with plaque vs. 50 % + site with plaque	0.56	0.36-0.87		/	/		/	/	
BOP			0.052			0.105			0.106
<25 % site with bleeding vs. 25 % + site with bleeding	/	/		/	/		/	/	
PPD			0.332			0.582			0.961
<25 % site with PD > =4 vs. 25 % + site with PD > =4	/	/		/	/		/	/	
CAL			0.204			0.107			0.672
0 % site with CAL > =4 vs. 0 % + site with CAL > =4	/	/		/	/		/	/	
Model 2									
Trimesters						0.960			0.690
First vs. Second				/	/		/	/	
vs. Third				/	/		/	/	
Previous births						0.879			0.068
None vs. One or more				/	/		/	/	
Utilization of dental services						0.006			0.044
No vs. Yes				2.52	1.30-4.88		2.07	1.02-4.21	
Frequency of nausea-vomiting						0.103			0.101
Once or less vs. Twice				/	/		/	/	
vs. Three times or more				/	/		/	/	
Self-repored systematic disease						0.152			0.172
No vs. Yes				/	/		/	/	
Model 3									
Age (Years)									0.009
25 or below vs. 26-30							1.71	0.97-3.02	
vs. 31-35							1.49	0.68-3.25	
vs. 36 or above							5.06	1.93-13.3	
Location									0.124
Urban vs. Rural							/	/	
Marital status									0.996
Married vs. Single							/	/	
Birth place									0.065
Shanghai vs. Other places							/	/	
Education level									0.244
Higher secondary school or below vs. Matriculation or bachelor degree or above							/	/	
Monthly household income									0.283
Less than RMB 6000 vs. RMB6000 or more							/	/	
Coverage of dental insurance									0.732
No vs. Yes							/	/	
Tooth loss									0.011
0 teeth missing vs. 1+ teeth missing							1.85	1.15-2.96	

^a^Likelihood Ratio *χ*2 = 11.4, df = 4, *p* =0.023; Deviance ratios = 1.02

^b^Likelihood Ratio *χ*2 = 22.2, df = 11, *p* =0.023; Deviance ratios = 0.99

^c^Likelihood Ratio *χ*2 = 49.5, df = 22, *p* =0.001; Deviance ratios = 0.96

Model 1: Poisson regression for periodontal condition variables

Model 2: Poisson regression for periodontal condition variables after adjustment for pregnancy-related variables

Model 3: Poisson regression for periodontal condition variables after adjustment for pregnancy-related variables and possible confounders

Logistic regression model (Models 1–3) for the prevalence of negative impacts (‘fairly often’ or ‘very often’) are presented in Table 8. In mode 1, BOP was significantly associated with prevalence of negative impacts. In mode 2, BOP and CAL was were found to be significant after adjustment for pregnancy-related variables. However, when adjustment for possible confounders, all these significant associations were no longer observed and no significant variables was found to be related with prevalence of negative impact (all p > 0.05).

**Table 8:** Results of Logistic regression for the prevalence of negative impacts (‘fairly often’ or ‘very often’) on the studied pregnant women

Variables	Model 1^a^			Model 2^b^			Model 3^c^		
	OR	95 % CI	P value	OR	95 % CI	P value	OR	95 % CI	P value
Model 1									
VPI			0.102			0.137			0.125
<50 % site with plaque vs. 50 % + site with plaque	/	/		/	/		/	/	
BOP			0.034			0.035			0.059
<25 % site with bleeding vs. 25 % + site with bleeding	2.13	1.06-4.28		2.16	1.06-4.44		/	/	
PPD			0.939			0.735			0.740
<25 % site with PD > =4 vs. 25 % + site with PD > =4	/	/		/	/		/	/	
CAL			0.070			0.040			0.262
0 % site with CAL > =4 vs. 0 % + site with CAL > =4	/	/		2.01	1.03-3.93		/	/	
Model 2									
Trimesters						0.990			0.982
First vs. Second				/	/		/	/	
vs. Third				/	/		/	/	
Previous births						0.448			0.837
None vs. One or more				/	/		/	/	
Utilization of dental services						0.134			0.131
No vs. Yes				/	/		/	/	
Frequency of nausea-vomiting						0.167			0.163
Once or less vs. Twice				/	/		/	/	
vs. Three times or more				/	/		/	/	
Self-repored systematic disease						0.467			0.504
No vs. Yes				/	/		/	/	
Model 3									
Age									0.913
25 or below vs. 26-30							/	/	
vs. 31-35							/	/	
vs. 36 or above							/	/	
Location									0.733
Urban vs. Rural							/	/	
Marital status									0.666
Married vs. Single							/	/	
Birth place									0.264
Shanghai vs. Other places							/	/	
Education level									0.747
Higher secondary school or below vs. Matriculation or bachelor degree or above							/	/	
Monthly household income									0.320
Less than RMB 6000 vs. RMB6000 or more							/	/	
Coverage of dental insurance									0.895
No vs. Yes							/	/	
Tooth loss									0.409
0 teeth missing vs. 1+ teeth missing							/	/	

^a^Cox & Snell R^2^ = 0.02, Nagelkerke R^2^ = 0.04

^b^Cox & Snell R2 = 0.03, Nagelkerke R2 = 0.06

^c^Cox & Snell R2 = 0.04, Nagelkerke R2 = 0.09

Model 1: Poisson regression for periodontal condition variables

Model 2: Poisson regression for periodontal condition variables after adjustment for pregnancy related variables

Model 3: Poisson regression for periodontal condition variables after adjustment for pregnancy related variables and possible confounders

## Discussion

Pregnancy is a significant period in a woman’s life; oral health care during pregnancy is an essential part of prenatal care, which is related not only to maternal health and well-being but also to the general health of the foetus. This study is the first to describe the OHRQoL of pregnant women in China by conducting an oral health survey with the Chinese version of OHIP-14. Good reliability and validity of the Chinese version of OHIP-14 were found.

In the present study, it was found that the mean number of the items reporting ‘fairly often’ or ‘very often’ (extent) was 0.20 with a mean OHIP-14 score of 7.92 (severity). Due to the lack of data pertaining to OHRQoL outcomes of nonpregnant women in Shanghai or from a national survey in the same age and gender, such comparison cannot be made. The mean OHIP-14 score (severity) from the present study (7.9) was similar to that of the pregnant women from rural India (7.0) [21], but was relatively higher than that of pregnant women from Brizal (3.8) [30].

The four items with the highest proportion of pregnant women reporting negative impacts were “taste worse”, “painful aching”, “uncomfortable to eat” and “self-conscious”; all of these items are within the domains of functional limitation, physical pain and psychological discomfort. Consistent with studies conducted amongst Hong Kong Chinese adults, the majority of Chinese populations showed that the impacts are within the same common domains, particularly functional limitation and physical pain [25, 31]. In a research conducted in India, the common impacts experienced by pregnant women are from the domains of physical pain and physical disability [20]. A study carried out amongst pregnant women from socially deprived populations in Argentina reported that the most frequent impacts were in the domains of psychological discomfort, functional limitation and physical pain when the OHRQoL was measured using OHIP-49 [22]. Nevertheless, physical pain is a common domain eliciting the most severe negative impacts on all ethnic groups. In pregnant Brazilian women with low income, oral pain (39.1 %), headache (61.5 %), pelvic pain (60.9 %) and back pain (59.3 %) have been observed [19]; furthermore, oral pain exhibits as much impacts on the daily lives of these women as back pain and pelvic pain [19]. However, oral pain is not entirely related to pregnancy and is avoidable to some extent. In contrast to oral pain, back pain and pelvic pain are considered as typical gestational symptoms because of physiological changes that women experience during this period.

Three scoring formats (severity, extent and prevalence) were adopted to assess the level of impact on quality of life in the present study. The severity (a simple summation of the response codes to all 14 items) of impact represents the overall burden of oral problem [32]. However, a related problem with severity scores is that a given score can be generated from different sets of responses with different items affected to a varying degree, therefore making it impossible to provide one ‘profile’ for a specific score [29]. For example, an OHIP-14 score of 7 may derived from a participant who may have many low-scoring impacts (7 items with ‘1’ and 7 items with ‘0’), or another participant who may have a few high-scoring impacts (1 item with ‘4’, 1 item with ‘3’ and 12 items with ‘0’). However, these participants are treated as being the same for analytic purposes but they have very different response profiles. In order to address this issue, different scoring formats (extent and prevalence) have been recommened and reported in some studies [16, 33]. The extent of negative impact refers to the presence and the number of problems with negative impacts. The prevalence of negative impact only indicates the presence of the problems with negative impacts. The use of three scoring formats in this paper would provide different perspectives in OHRQoL and the associated factors observed. In the present study, according to the results from multivariable analyses, it was found that periodontal conditions was not significantly associated with three scoring formats of OHRQoL after adjustment for pregnancy-related variables and possible confounders. However, frequency of nausea-vomiting was found to be significantly associated with severity of impacts (the overall burden of oral problem). Utilization of dental services, age and tooth loss were the significant variables to the extent of negative impacts (‘fairly often’ or ‘very often’), that is, the presence and the number of problems with negative impacts. While no siginificant variables was found to be related with prevalence of negative impacts (‘fairly often’ or ‘very often’) which refers to the presence of the problems with negative impacts.

There is a consensus that periodontal disease is considered as the most prevalent oral disease amongst pregnant women; the maintenance of oral health may prevent adverse pregnancy outcomes [13, 14]. Although periodontal health status was assessed in the present study by using BOP, PPD and CAL with full-mouth examination, no significant association was found between three scoring formats of OHRQoL and periodontal health status. Consistent with the findings from the study conducted amongst pregnant women in Uganda, periodontal health status, assessed using CPI, also has no impact on OHRQoL [18]. However, in the study conducted amongst Indian pregnant women, significant correlation between OHRQoL and CPI has been observed when bivariate analysis is performed [20], which is the case in the present study. In the present study, significant associations were found in bivariate analysis, but these associations were no longer observed when the parameters were adjusted for pregnancy-related variables and possible confounders. Although a number of periodontal pocket and clinical attachment loss were observed in pregnant women, other more severe health problems, such as nausea-vomiting, may be encountered by women during pregnancy. Thus, other severe health problems may become their major concern, which may impact their quality of life.

From the result on multivariable analyses, poorer OHRQoL (higher severity socre of impact) was observed in pregnant women who suffered from severe nausea-vomiting frequency. The frequency of nausea-vomiting amongst pregnant women is significantly related to severity of impact in the present study. Nausea and vomiting during pregnancy are common health problems that affect up to 90 % of pregnant women [34]. Nausea-vomiting also elicits a pervasive detrimental impact on women’s family, social and professional lives [35]. However, common treatments and standard advice may not be effective and rarely provide complete relief [36]. Hence, further studies involving physiological and psychological mechanisms should be conducted to elucidate the occurrence of nausea and vomiting during pregnancy and help alleviate this problem.

It is disappointing that only 1.2 % of the pregnant women utilised dental services for regular dental checkup during pregnancy in the present study. This finding is significantly lower than that in the United States (49 %) [37]. Likewise, a noticeable proportion of pregnant women in Brazil did not seek dental treatment to relieve pain; most of these pregnant women believed that dental treatment should be avoided during pregnancy because they consider the safety of the foetus and assume that oral pain is normal during pregnancy [19]. A similar belief of “one tooth, one child” is also widespread amongst pregnant Ugandan women; thus, approximately three quarters of pregnant women have never sought dental treatment [18]. The same belief of Brazilian and Ugandan women is probably held by Chinese women. In the present study, it is not surprising to find that pregnant women who utilised dental services had significantly higher extent of negative impact than those who did not employ such services according to the result on multivariable analyses. Problem-oriented dental visit pattern was observed amongst pregnant women in Shanghai because the majority of pregnant women who underwent dental visits suffered from severe oral problems. These severe oral problems affect OHRQoL; thus, the relationship between utilisation of dental service and OHRQoL can be easily understood. In Shanghai, free antenatal health care education programs are provided for pregnant women once they attend antenatal checkup in maternal and child care service centres. However, these programs emphasize very little on oral health care during pregnancy and the safety of dental care for pregnant women. In order to improve maternal health and well-being, the alternative strategy may be to integrate the oral health promotion or education into already existing antenatal health education programs. Moreover, future antenatal health education programs may include oral health care professionals to deliver oral health education to investigate the effectiveness of oral health education programme.

In a systematic review, tooth loss, the true endpoint of dental caries and periodontal disease, possibly elicits a negative impact on OHRQoL [38]. In a study amongst pregnant women in Uganda, a significantly strong association was observed between OHRQoL and tooth loss. Moreover, tooth loss mainly leads to dental functioning impairment, but dental appearance and social concerns are less important, specifically in young age groups [18]. In the present study, it was found that tooth loss was significantly associated with the extent of negative impacts. This finding are consistent with those in previous studies even if only less than one-fifth of the pregnant women experienced tooth loss. The low prevalence of tooth loss may be due to the age distribution of the study sample with a median age of 27 years; furthermore, tooth loss is a common phenomenon in Chinese population aged >40 years [39].

## Conclusions

Pregnant women with different trimesters experienced a similar impact of oral disease on their OHRQoL in Shanghai, China, as determined using OHIP-14 as a quality of life measure. The negative oral impacts experienced by women were mainly in the aspects of functional limitation and physical pain. Periodontal health status have no impact on their OHRQoL in the fully adjusted models. Their OHRQoL was associated with early pregnancy reaction, utilisation of dental services, age and tooth loss.
